# Cell Metamorphosis

**Published:** 1893-09

**Authors:** P. T. Smith

**Affiliations:** Denver, Col.


### CELL METAMORPHOSIS.[1]

BY P. T. SMITH, D.D.S., DENVER, COL.

Cell-growth is recognizable in all vegetable and animal organisms, there being as many different kinds of cells as there are specific or even intimated characteristics in the body.

The primary inception of a living body is but a single cell, fertilized.

---

[1] Read before the Colorado State Dental Association, June 7, 1893.

The myriad of elements that enter into the composition of a primate cell and reflect substantial evidence of their existence and power in the ultimate, do so by means of a specific class of cells, —each of a determinate idiom, and which proliferate from the parent cell. To follow the cell quality throughout the differentiation necessary to crystallize and personate each factor of the whole organism would create a volume. Hence I can but hint at some salient ideas bearing upon the functions and metamorphoses of some of those most familiar.

Commonly, as we understand it, cells are primarily created out of protoplasm, and consist of a body, nucleus, and nucleolus. This analysis will not sufficiently account for the heredity or transmission of parental idiosyncrasies which involve phenomena of much importance to the pathologist.

Germ-plasm differs from protoplasm in being active, while the latter is a passive substance and a cell-culture susceptible of inhibition in the growth and division of the nucleus, and endowed with biophors under control of the determinates or ids of Weismann, or gemmules of Darwin. These substances are alone formed in the nucleolus; the ids or gemmules controlled by the determinates, and in the exhibition of sperm-plasm fertilizing the ova-plasm, there comes from every department of the paternal and maternal source of authority a biophore or id which bears the nature of its origin to the new objective independent, impressing the new being with mental and physical likeness of its combined parental origin.

These functions, with the associated ones of sensation and movement, are connected in all organisms with which we are familiar, from the simplest univalvular forms to the highest plants and animals, with at least two different substances: the idioplasm of the nucleus, or the hereditary-plasm in the more general sense, and protoplasm of the cell-body. These two differ as regards their functions, though they resemble each other in being composed of living substance.

Nageli's example is acceptable in calling the vital substance of the cell the "formative plasm or morphoplasm" in contrast to the idioplasm. The latter is the active element in the process of formation, and the former the passive one. As we now believe that the idioplasm is situated in the nuclei only, we cannot regard the cell-bodies which determine the form of all parts of the organism as mere nutrient plasm.

The germ-plasm contains the primary constituents of all the cells in the body in its determinants, which, in their highly-endowed susceptibility, are subject to the varied controlling influences reflected from compatible as well as incongruous and degenerate sources, such as parental predominance in mental and physical inhibition as well as extraneous involutionary nutrients,—*i.e.*, vital currents, food, air, and water.

Cells composed of physiological elements, designed by the chemical proportions for specific functional service, will evolve a faithful reflex of the combined parental image setting forth each individuality of the minutest architecture, faithful in the most scrupulous degree. This is recognizable only under highly favorable conditions in the complicated phenomena of ontogeny.

Again, as these supersensitive functional centres are ever subservient to the behests of any control, how great the danger from the secret menace of pathological forces, ready to impose an abiding condition at this incipient period of cell-life that will inhere throughout all subsequent proliferation to the ultimate formation within the function of this particular cell individuality!

Scrofulous taints, chalky and syphilitic teeth, are evolved in this way. Deformed and degenerate mouths, as well as every other inherited pathological condition, are the result of metamorphosed or perverted cell-action.

Cell-action or function is invariably dependent upon composition, quantity, and kind for the part it plays in the economy.

The osteoclastic cell which arises from the white blood-corpuscles does so only by a metamorphosic action, which involves chemico-organic change.

The odontoblast, which in the primate architecture of ontogeny was normal and endowed with the ids and determinants of full power, may, under the appalling influence of a fever or cold or malarial poison, mercurial or other mineral medicinal toxicants, inhibited in early formation of life, be so changed or wrought upon as to show in its work an inefficiency hardly recognizable as compared to the pleasing result of uninterrupted, harmonious functional action.

The nutrient supply and growth is maintained from the cell-plasm substance, attracted by the alternative positive and negative conditions subservient under the immutable law of involution.

